# Beyond evidence accumulation: shared-goal belief guides action generalization in social groups

**DOI:** 10.1186/s41235-025-00666-x

**Published:** 2025-08-26

**Authors:** Jipeng Duan, Yinfeng Hu, Wenying Zhou, Qingqing Ye, Ting Zhao, Jun Yin

**Affiliations:** 1 Department of Psychology, Ningbo University, No. 616 Fenghua Rd., Ningbo, 315211 China; 2 Center of Group Behavior and Social Psychological Service, Ningbo University, Ningbo, China

**Keywords:** Social group, Action generalization, Belief, Evidence, Shared goal

## Abstract

People tend to generalize the actions of known group members to new ones when they are both members of the same group. This study was conducted to investigate how the prevalence of specific actions among multiple individuals determines action generalization within social groups. We propose that people rely on the belief that group members work toward a shared goal (i.e., shared-goal belief) to guide action generalization. Consequently, the extent of action generalization may not consistently increase with the sampled prevalence of group members performing the same goal-directed action, resulting in a deviation from graded action generalization (i.e., nongraded action generalization). Experiment 1 revealed that the more participants believed that group members pursued a shared goal, the greater the likelihood that nongraded action generalization would occur. In Experiment 2, experimental manipulation weakened the strength of the shared-goal belief and led to a graded pattern of action generalization with accumulated evidence of action prevalence. These findings suggest that a shared-goal belief within groups significantly shapes action generalization beyond the mere influence of sampled action prevalence. Social groups not only provide a framework for selecting evidence for action generalization but also shape prior beliefs that influence our expectations of others’ actions.

**Supplementary Information:**

The online version contains supplementary material available at 10.1186/s41235-025-00666-x.

## Significance statement

Individuals exhibit a tendency to generalize actions from a familiar individual to a new individual if both belong to the same social group. This study highlights that, beyond the evidence of prevalent actions gathered, the default belief in shared group goals plays a significant role in action generalization. Consequently, when this belief is present, the extent of action generalization does not consistently increase in direct proportion to the prevalence of goal-oriented actions, indicating a nongraded pattern of action generalization based on accumulated evidence. These findings suggest that social groups not only establish limits for selecting action-related evidence but also mold prior knowledge that shapes our expectations of others’ actions. This study reveals that individuals intuitively understand group dynamics, although this capacity varies across contexts and individuals, even without specific knowledge of the group’s attributes. In practice, managers can leverage a shared-goal belief to promote expectations of group-consistent behaviors, complementing efforts to increase the prevalence of majority behaviors within teams or organizations.

**Supplementary Information:**

The online version contains supplementary material available at 10.1186/s41235-025-00666-x.

## Introduction

People’s social lives are closely tied to their expectations of how others will behave. However, this process poses a significant challenge when determining how past experiences and available knowledge about individuals can predict future encounters: the problem of inductive generalization (Andrews, 2008; Kalish, 2012). Although previous actions of an individual can serve as a helpful indicator of their behavior in similar situations, there are instances when we may lack sufficient knowledge about the individual to form expectations about their actions (Bodenhausen et al., 2012; Vijayakumar et al., 2021). In such cases, we often turn to our knowledge and experience of social groups to support our expectations of others’ actions.

Research suggests that people tend to generalize the actions of a known individual to a new one when they are both members of the same group, which is known as category-based induction (Grigoryan, 2020; Hu et al., 2023; Yin et al., 2023). The problem of generalizing actions within social groups involves three elements. Actions performed by a subject may be extended to a target as a member of the same social group. For example, if John takes an apple instead of a banana and we observe this action previously, we expect that James from the same group as John will also take an apple. In this case, John is the subject, taking an apple is the predicate, and James is the target. Multiple subjects of generalization can exist within a social group, and the process becomes more complex when the subjects do not consistently exhibit the same behaviors or actions. How does the prevalence of specific actions among multiple subjects determine action generalization within social groups? The current study demonstrated that this generalization is not only driven by the observed action prevalence but also shaped by the belief that group members work toward a shared goal.

### Action generalization within group members

Humans strongly depend on social groups for social connection and collective action, which has shaped the evolution of our cognitive abilities to process and represent social groups (Hirschfeld, 2001). Social groups usually indicate that individuals who share group membership are fundamentally similar to one another (Billig & Tajfel, 1973; Bodenhausen et al., 2012; Macrae & Bodenhausen, 2000). Consequently, both adults and infants generalize the actions and attributes of known members to unknown or new members within the group (i.e., action generalization), resulting in the expectation that all group members will act similarly (Powell & Spelke, 2013; Ranganath & Nosek, 2008; Yin et al., 2022, 2023).

Recent studies have provided more direct evidence of action generalization among group members. Specifically, after observing two ingroup members’ actions, participants identified group-consistent actions faster than group-inconsistent actions, indicating action generalization from known members to new ones, as the group-consistent actions aligned with their expectations about group members’ similar behaviors (Duan et al., 2021; Xu et al., 2019). Moreover, actions are typically directed toward external targets or goals, known as object-directed actions (Phillips & Wellman, 2005). Such actions can be represented as possessing both the property of movements and the property of action goals (Cavallo et al., 2013; Csibra, 2008). An action goal refers to an external target (e.g., an apple), while movement refers to the means used to achieve the goal (e.g., jumping vs. moving straight). For example, if an agent jumps instead of moving straight to approach an apple, the jumping is the movement, and reaching the apple is the goal. Although both the action goal and movement can be treated as generalized properties, people primarily generalize action goals rather than superficial movements among members of a social group (Yin et al., 2022, 2023). This generalization effect is based on the multiple sampled group members, with the majority performing the same action rather than a single group member repeating the same action. Hence, social groups provide a framework for selecting evidence for action generalization.

As a form of inductive generalization, the extent of action generalization within social groups is typically influenced by the sampled prevalence of relevant actions among observed group members (Hayes et al., 2019; Tentori et al., 2016). For example, the proportion of group members approaching an apple rather than a banana often determines the likelihood of generalizing this action to others. Hence, there should be a graded (or monotonic) relationship between the prevalence of a target action and its generalization: The more that group members exhibit the target action, the greater the likelihood that this action will be generalized to others, and vice versa. However, research has shown that action generalization does not always increase proportionally with the prevalence of the target action. Specifically, when all observed group members pursue the same goal, the target action is largely generalized to a new group member in a nongraded manner (Hu et al., 2023). This finding indicates that action generalization is shaped not only by observed evidence but also by additional factors.

Previous studies have indicated that inductive generalization is largely influenced by prior knowledge of the properties being generalized (Kalkstein et al., 2020; Lazaridou-Chatzigoga et al., 2019; Voorspoels et al., 2015). For instance, in a male-dominated context, the performance of a female leader is more likely to be attributed to a female candidate for a leadership position, whereas no such effect is observed for male leaders (Manzi & Heilman, 2021). Such studies have primarily examined how prior knowledge about specific attributes—such as gender stereotypes—influences patterns of generalization (Hayes et al., 2003; Klaczynski et al., 2020). However, individuals also possess more general knowledge about social groups and an intuitive understanding of group dynamics, including prior beliefs about how group members are expected to behave, even before encountering new information or evidence (Chalik & Dunham, 2020; Chalik et al., 2014; Hu et al., 2023; Rhodes & Chalik, 2013). One such belief is that group members tend to pursue a shared goal (Brewer et al., 2004; Noyes & Keil, 2020). Nonetheless, relatively little research has investigated whether such group-related beliefs constrain action generalization.

### Beliefs about how group members act

Given that social groups play a vital role in our survival and shape our social abilities in terms of how we perceive and interact with others, it is suggested that individuals possess an intuitive understanding of social groups. This understanding involves preconceived beliefs about how group members are likely to act before any new information or evidence has been presented. For example, studies have revealed that a belief of interpersonal obligation exists whereby people, including children, believe that members within a group should not harm one another (Chalik & Dunham, 2020; Chalik et al., 2014; Rhodes & Chalik, 2013).

In this paper, we propose the belief that group members behave toward a shared goal. This belief may be rooted in the basic function of the group, particularly for social groups formed to accomplish a task. Social groups are organized to address coordination problems, and they function by working together to achieve collective goals, such as a group of individuals playing football. Shared goals facilitate smooth interactions (Sebanz et al., 2003) and constrain individuals’ actions within the group (Kachel & Tomasello, 2019). Moreover, a group’s function determines its existence and continuity, and changing the group’s realized function or goal can challenge its sense of continuity, even if group members are recognized as being similar (Guala, 2016; Noyes & Keil, 2020). Social groups can be perceived as agentic actors with a strong sense of agency that can achieve a desired end state (Bloom & Veres, 1999; Cooley et al., 2017; Sheikh & Hirschfeld, 2019). In this interpretation, group agency is characterized by coordination to achieve a shared goal rather than the mere similarity of behavior (Brewer et al., 2004).

Accordingly, instead of delineating the boundary for selecting evidence during action generalization, social groups identify members as pursuing a shared goal by default. In such instances, this prevailing belief might constrain the impact of accumulated evidence on how actions are generalized among group members. However, few studies have examined this speculation. The current study aims to explore whether a shared-goal belief within a group plays a role in action generalization. A positive finding suggests that people possess an intuitive understanding of social groups and hold prior beliefs about how group members should behave that even dominates the strength of observing any actual action.

### Overview of the current study

**Fig. 1:**
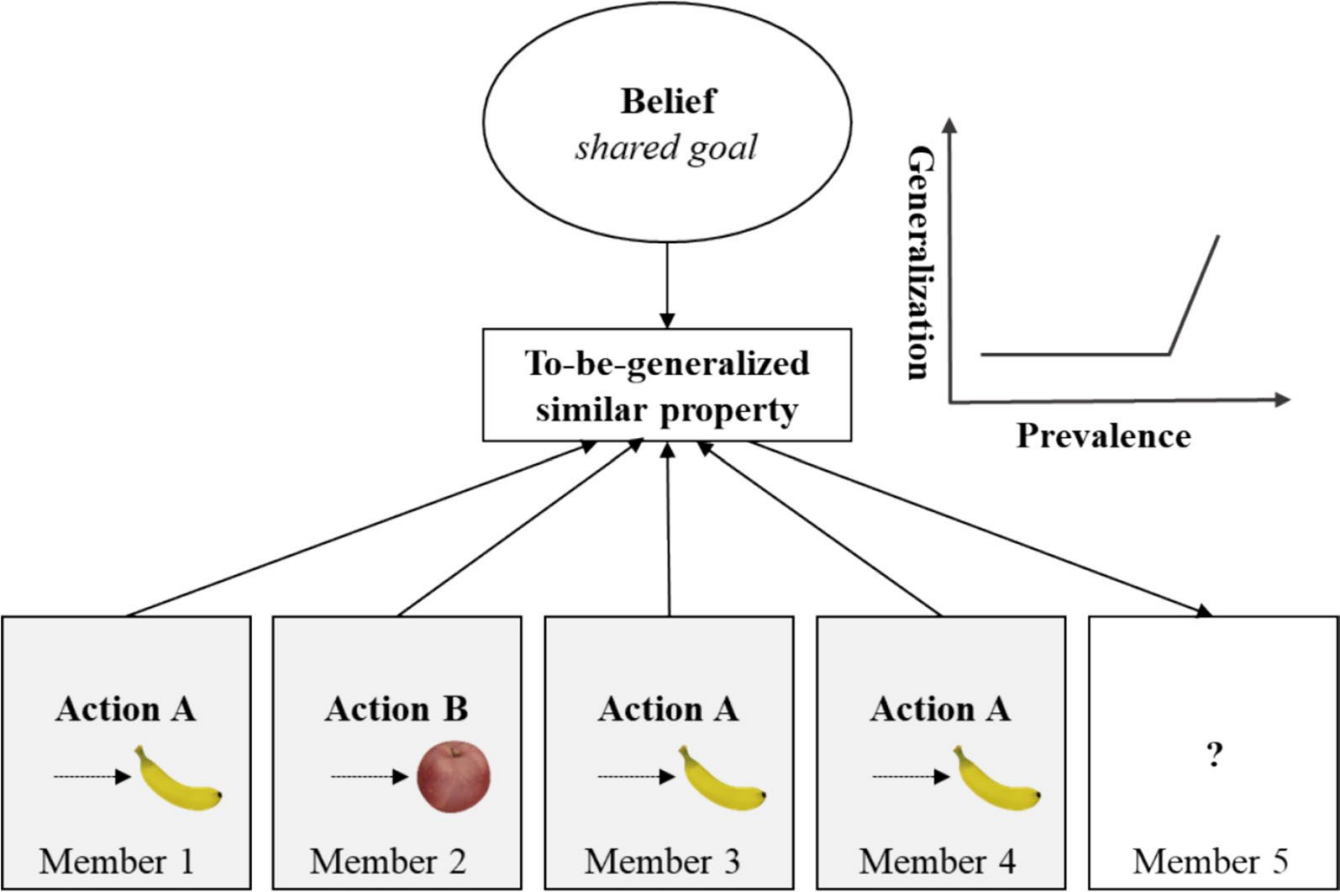
Illustration of how shared-goal belief influences the relationship between action prevalence and the extent of action generalization

In this study, we propose that social groups shape prior beliefs about shared goals, which in turn influence our expectations of individual actions within the group (Note that the term “action” here specifically refers to performing an object-directed action). When a group member is observed acting toward a different goal, this action conflicts with the shared-goal belief, and a discrepancy arises between the shared-goal belief and the observed action, which makes it unclear whether similar actions should be generalized. As a result, even if the statistical prevalence of a particular action increases, the shared-goal belief limits the extent to which the action is generalized across members. However, when all known group members pursue a common goal, this observation aligns well with the shared-goal belief, and this specific action is considered a similar property to be extended to unknown group members. In essence, when a shared-goal belief is present, the strength of the action generalization depends more on goal consistency within observed actions than on action prevalence alone. Therefore, action generalization is not strictly graded on the observed evidence of action prevalence when prior beliefs about shared goals within social groups are available. That is, individuals do not incrementally change their generalizations in line with increasing prevalence. Instead, generalization may plateau or remain unchanged despite more supporting evidence (see Fig. 1), reflecting a deviation from graded generalization—referred to here as “nongraded” for brevity.^1^ However, when the beliefs concerning shared goals among social groups are reduced or inaccessible, the process of action generalization can predominantly hinge on sampled evidence. Consequently, as the quantity of evidence sampled increases, the extent of action generalization also increases and displays a graded pattern of action generalization (referred to as the shared-goal effect).

Therefore, this study aims to demonstrate that prior beliefs can qualitatively alter the pattern of generalization as evidence increases rather than defining the precise functional form of action generalization (e.g., linear vs. nonlinear) based on observed evidence. When generalization fails to increase despite accumulating evidence—a pattern that we refer to as nongraded—it highlights the influence of the shared-goal belief. The shared-goal effect has been previously observed by manipulating the action prevalence of group members approaching an external target. The results showed that the extent of action generalization does not significantly differ when half of the group members approach the same goal versus when three-quarters approach the same goal (Hu et al., 2023). However, previous studies have not established the direct and causal contribution of the belief in group members having shared goals to the shared-goal effect because of the lack of measurement and manipulation of this belief. A complete test of our hypothesis should examine what occurs when the belief in group members having shared goals is reduced or inaccessible. In such situations, a rise in action prevalence will strengthen the individual’s tendency to extend this action to unknown group members, as emphasized in inductive generalization (Hayes & Heit, 2018). Consequently, when the shared-goal belief is absent, people rely solely on observed evidence. As the proportion of group members pursuing a common goal increases, they are more likely to assume that an unknown group member will exhibit this action, resulting in a graded pattern based on action prevalence.

The present study involved two experiments that investigated the potential contribution of the belief regarding shared goals among group members to action generalization, specifically, whether it occurs in a nongraded manner. In Experiment 1, the action prevalence was manipulated by varying the proportion of group members approaching the same external target. Belief ratings regarding shared goals among group members and action generalization ratings under different prevalence rates were collected to explore the correlation between shared-goal belief and nongraded action generalization. Experiment 1 was a correlational study; thus, Experiment 2 was conducted to provide causal evidence by presenting different types of groups and textual materials describing how group members behave, thereby manipulating the strength of the shared-goal belief among group members. Our hypothesis predicted that when the shared-goal belief is greater, the nongraded action generalization will be greater and that when this belief is weakened through manipulations (i.e., different types of groups and textual materials), graded action generalization will be observed.

## Experiment 1

Through the use of a questionnaire survey, this study investigated the relationship between the belief that group members share goals and the pattern of generalizing actions among group members. Specifically, if the shared-goal belief is utilized for action generalization, then the stronger this belief is, the stronger the nongraded generalization between the extent of action generalization and the action prevalence will be.

### Methods

#### Transparency and openness

We reported all measures, manipulations, and exclusions (if any) for all of the experiments and followed the journal article reporting standards (JARS) (Kazak, 2018); however, none of the study designs were preregistered. All data, analysis codes, and example trial videos for each condition have been made publicly available on the OSF platform and can be accessed at https://osf.io/6ap74/?view_only=97741fd0566a41c49378f582e740abb7. We analyzed the data using R version 4.1.2 with the bruceR library version 0.8.9 (Bao, 2022) and the ggplot2 package version 3.1-3 (Wickham, 2011).

#### Participants

We recruited 110 participants from multiple universities to increase the diversity and inclusiveness of our sample in terms of participant demographics. All participants were of Han ethnicity, the majority ethnic group in China. After completing the investigation, the participants were compensated approximately 8 RMB each. We used G*Power 3.1 (Faul et al., 2009) to determine the sample size, with an α level of 0.05 and power of 0.80, and we assumed a medium effect size of *r* = 0.3 for significant correlation detection. The suggested minimum sample size was 84; however, we included 110 participants to be conservative and ensure sufficient valid data. Seven participants were excluded because of incomplete investigations, leaving 103 valid participants (43 men and 60 women, *M*_age_ = 19.89 years, SD = 1.73) for the analysis. A sensitivity power analysis indicated that this sample size provided 80% power to capture a medium effect of correlation (*r* ≥ 0.27).

The study was approved by the Research Ethics Board of the Department of Psychology at the authors’ university and was performed in accordance with the relevant guidelines and regulations. For all of the experiments, we reported all measures, manipulations, and exclusions.

#### Procedure and design

This experiment comprised two phases: The first involved measuring the shared-goal belief, while the second focused on measuring action generalization.

*Measuring Shared-Goal Belief* This phase presented the video separately from the beginning of the phase that measured action expectation; the video displayed how the social group was formed, resulting in two videos with two types of targets (i.e., food and drink; for the demonstration videos, please refer to OSF link https://osf.io/6ap74/?view_only=97741fd0566a41c49378f582e740abb7). In one video, ten individuals were situated in a room with six apples and six bananas placed on separate tables a short distance away from them. The ten individuals were divided into two social groups of five, identified by matching waistcoats, and were seated at opposite corners of the room. In addition, individuals wearing the same-colored waistcoat interacted with one another to emphasize their social group identity (Ip et al., 2006). In another video, the food items were replaced with drinks, namely Cola and Sprite. After watching each video, the participants were asked to rate their beliefs about the group’s shared goals using a five-item scale: “How likely is there commonality of goals among the group members wearing the same waistcoat?”, “How likely are the group members wearing the same waistcoat to pursue collective goals?”, “How likely are the group members wearing the same waistcoat committed to pursuing collaborative goals?”, “How likely are the group members wearing the same waistcoat to view themselves as partners in charting the project directions?”, and “How likely are the group members wearing the same waistcoat to be in total agreement on their project work?” The ratings were determined on a scale ranging from 1 (completely unlikely) to 7 (completely likely). The internal consistency of these items was estimated via Cronbach’s alpha, which was 0.78 in Experiment 1. The items were adapted from previous research (Chow & Chan, 2008; Nadeem et al., 2021) to suit the current scenarios.

*Measuring Action Generalization* The participants were shown a sequence of six videos featuring two groups of five individuals at a party who were tasked with retrieving food or drinks. The videos used to measure action generalization included the same group of people as those used to measure the shared-goal belief. After each video, the participants were asked two questions. To ensure diversity, two different party scenarios were presented. In one scenario, ten individuals were situated in a room with six apples and six bananas placed on separate tables at a short distance from them. The ten individuals were divided into two social groups of five, identified by matching waistcoats, and were seated at opposite corners of the room. At the beginning of the video, the group members interacted with one another to emphasize their social group identity. Subsequently, four individuals from the same group proceeded individually to the front of the room and then turned left or right to retrieve food from one of the tables. The action prevalence of food selection varied among the videos. In the high prevalence condition, four individuals chose the same food item, whereas in the medium prevalence condition, three individuals chose the same item, while the fourth chose the alternative. In the low prevalence condition, two individuals chose each food item. After their food was retrieved, the individuals returned to their original seats. A new group member then approached the table, faced the participant, and paused. The participants were asked two questions—“How likely is this person to take an apple?” and “How likely is this person to take a banana?” (rated from 1 = completely unlikely to 7 = completely likely)—to avoid measuring only a specific type of individual goal-directed action and recognizing that the other action may also be generalized. In a separate scenario, the food items were replaced with drinks, namely Cola and Sprite. Each participant watched a total of six videos, with two videos representing each prevalence condition. The presentation order in which group members pursued a different goal was randomized across the participants.

The action generalization index was constructed to measure the participants’ expectations about whether the fifth person would perform the prevalent action. The index was computed by averaging the mean ratings of both the food and drink scenes (*α* = 0.60), with items related to the likelihood of taking the minority’s food or drink being reverse-coded. In the low prevalence condition (i.e., 2 out of 4 group members performing a particular action—A—but the other two performing another action—B), which represents an equal prevalence for each action, the prevalent action is randomly determined. It aims to provide a computational logic that is consistent with that used in the medium and high prevalence conditions. This method has been used previously (Hu et al., 2023). Both actions in the low prevalence condition were found to be equally expected (for details, see the Supplementary Materials), justifying the random assignment of the prevalent action. For the computed index, the midpoint (i.e., 4) on the 7-point scale (1 = completely unlikely to 7 = completely likely) reflects participants’ uncertainty about which of the two distinct actions the individual will perform. When unsure, participants’ ratings are expected to center on the midpoint rather than the lowest value (i.e., 1), which would indicate confidence that the rated action will not occur and that the alternative action (i.e., take an apple) will occur.

The videos and rated items were presented using PsychoPy software. To familiarize the participants with the rating scale, they were first shown a weather report image and asked to rate the likelihood of the temperature rising on a scale from 1 (completely unlikely) to 7 (completely likely), which they indicated by using a mouse to mark the corresponding point on the screen. The order of the videos in each phase was randomized to prevent order effects. The entire experiment lasted approximately 10 min.

### Results and discussion

**Fig. 2:**
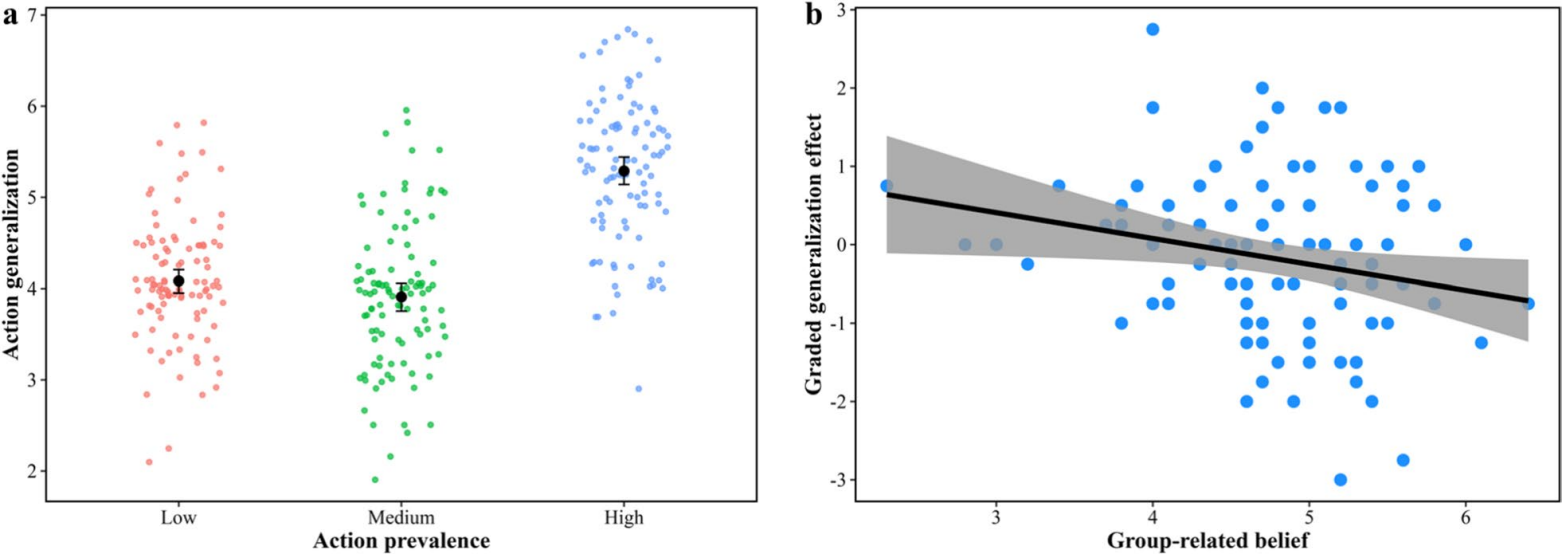
Action generalization as a function of action prevalence (**a**) and the correlation between the degree of shared-goal belief and the graded generalization effect (**b**). The large circles denote the group means in each condition, and the black vertical lines indicate the 95% confidence intervals (CIs) of the means (the same below). The gray region represents the 95% CI of predictions from a linear model

The mean action generalization index of each participant for each condition is shown in Fig. 2a. Notably, in the low prevalence condition, the rating value was not significantly different from the midpoint of the rating scale (i.e., 4) (*t*(102) = 1.24, *p* = 0.217, Cohen’s *d* = 0.12, 95% CI of *d* = [− 0.07, 0.32]), thus confirming the baseline for other conditions. A repeated-measures analysis was conducted on the extent of action generalization, with action prevalence (low vs. medium vs. high) as the independent variable. The results revealed a significant effect of action prevalence (*F*(2, 204) = 102.09, *p* < 0.001, *η*_p_^2^ = 0.50). Post hoc comparisons via Bonferroni correction indicated that the extent of action generalization in the high prevalence condition (*M* = 5.29, SE = 0.08) was greater than that in the medium (*M* = 3.91, SE = 0.08; *t*(102*)* = 12.35, *p* < 0.001, Cohen’s *d* = 1.29, 95% CI of *d* = [1.04, 1.55]) and low- (*M* = 4.08, SE = 0.07; *t*(102*)* = 12.09, *p* < 0.001, Cohen’s *d* = 1.13, 95% CI of *d* = [0.90, 1.36]) prevalence conditions but that there was no significant difference between the medium and low prevalence conditions (*t*(102*)* = 1.66, *p* = 0.298, Cohen’s *d* = 0.16, 95% CI of *d* = [− 0.08, 0.40]). Therefore, the extent of action generalization does not necessarily increase with the prevalence of observed action. Even as prevalence intensifies, when group members pursue inconsistent goals, action generalization does not increase.

We conducted further analyses to investigate whether the extent to which individuals believe that the group has a shared goal predicts the relationship between action prevalence and action generalization. We first calculated the degree to which the weak prevalence weakened the action generalization for both the low and medium prevalence conditions relative to the high prevalence condition. We then subtracted the degree of weakening for the medium prevalence condition from that of the low prevalence condition to obtain the graded generalization effect index. This index measures whether the extent of action generalization increases with the prevalence of observed action when not all group members approach the same goal (i.e., graded generalization effect). We found a significant negative correlation between the degree to which individuals believed that the group had a shared goal and the graded generalization effect (*r* = − 0.22, *p* = 0.028, 95% CI = [− 0.39, − 0.02]; Fig. 2b). This finding suggests that the more that individuals believe that group members have a shared goal, the lower the likelihood that they will generalize the prevalent action to a new group member. This finding offers initial evidence that individuals’ belief that group members share a goal determines how action prevalence influences action generalization.

## Experiment 2

The results of Experiment 1 were correlational and provided limited evidence for our hypothesis. Therefore, in two subsequent sub-experiments, we manipulated shared-goal belief using different methods to uncover experimental evidence that this belief contributes to the shared-goal effect.

In Experiment 2a, shared-goal belief was manipulated via different types of groups, specifically, dynamic and categorical social groups. Dynamic groups refer to interacting, socially connected individuals, whereas categorical groups refer to members of a category (Rutchick et al., 2008; Wilder & Simon, 2013). Previous studies have shown that people make a conceptual distinction between dynamic and categorical groups, also referred to as task groups and social-category groups, and hold different beliefs or ways of thinking about them (Lickel et al., 2000, 2006; Rutchick et al., 2008). Dynamic groups, formed through the pursuit of collective goals (Effron & Knowles, 2015), are more likely to elicit a strong belief in shared goals than categorical groups are (Rutchick et al., 2008; Spencer-Rodgers et al., 2007). If our experimental hypothesis is valid, then when the group is dynamically construed, the impact of action prevalence on expecting group members’ actions should show a nongraded pattern, while when the group is categorical, this pattern should disappear or weaken and become graded.

However, the inherent differences between group types—such as perceived entitativity or cohesion (Lickel et al., 2000, 2006; Rutchick et al., 2008)—might also affect action generalization independent of shared-goal belief. To account for this possibility, Experiment 2b used textual descriptions to directly manipulate the strength of shared-goal belief within the same group type (Henderson, 2009). If the hypothesis is supported, then a nongraded generalization pattern of the impact of action prevalence on expecting group members’ actions is more likely when the belief is described in the context of a strong tendency for shared goals among group members.

### Experiment 2a

#### Methods

A total of 149 participants participated in this study and were compensated approximately 5 RMB each upon completion of the investigation. All participants were of Han ethnicity, the majority ethnic group in China. The sample size was determined using G*Power 3.1 (Faul et al., 2009) with a power analysis that set the alpha level at 0.05 and the power at 0.80 and aimed to detect a difference with a small–medium effect size (Cohen’s *d* = 0.3 or *η*_p_^2^ ≈ 0.09) across the three action prevalence conditions. The minimum suggested sample size was 73 individuals for each between-subjects group, resulting in a total of 146 individuals for both groups. To ensure sufficient valid participants, 149 participants were recruited. However, two were excluded due to incomplete investigations, leaving a total of 147 valid participants (62 men and 85 women, *M*_age_ = 20.09 years, SD = 1.96) who were randomly assigned to two groups. Finally, 74 participants were included in the dynamic group condition, and 73 were included in the categorical group condition. A sensitivity power analysis indicated that this sample size provided 80% power to detect an effect with Cohen’s *d* ≥ 0.46 in different action generalization patterns between the two group types.

The study included two group types: dynamic and categorical. The videos for each group type varied in how the social group was introduced. In the dynamic group condition, the six videos were the same as those in the action generalization phase in Experiment 1. In the categorical group condition, at the beginning of each video, one group of five people wearing blue waistcoats stood up and said, “I am a member of the blue team,” and then sat down; next, another group of five people wearing red waistcoats stood up and said, “I am a member of the red team,” and then sat down. The order in which the groups stood up was counterbalanced. Therefore, the design of this experiment was 2 (group type as a between-subject factor: dynamic vs. categorical group) × 3 (action prevalence as a within-subject factor: low vs. medium vs. high). The action generalization index was computed by averaging the mean ratings of both the food and drink scenes (α = 0.84), with the items related to the likelihood of taking the minority’s food or drink being reverse-coded.

A new group of 103 participants (29 men and 74 women, *M*_age_ = 20.70 years, SD = 2.01) was recruited to validate the shared-goal belief manipulation in this experiment. This group watched segments of the videos displaying how the social groups were formed for each group type, similar to the shared-goal belief measuring phase in Experiment 1. Specifically, 51 participants watched dynamic group videos, and 52 watched categorical group videos. After watching each video, the participants were asked to rate their beliefs about the group’s shared goals using the same five-item scale (*α* = 0.75) as in Experiment 1.

#### Results and discussion

*Validity of Shared-Goal Belief* An independent *t* test revealed that the belief that group members pursue a shared goal was rated more strongly for the dynamic group (*M* = 5.30, SE = 0.09) than for the categorical group (*M* = 4.97, SE = 0.11; *t*(101*)* = 2.34, *p* = 0.021, Cohen’s *d* = 0.46, 95% CI of *d* = [0.06, 0.86]). This finding suggests that the manipulation of shared-goal belief in this experiment is valid.

**Fig. 3:**
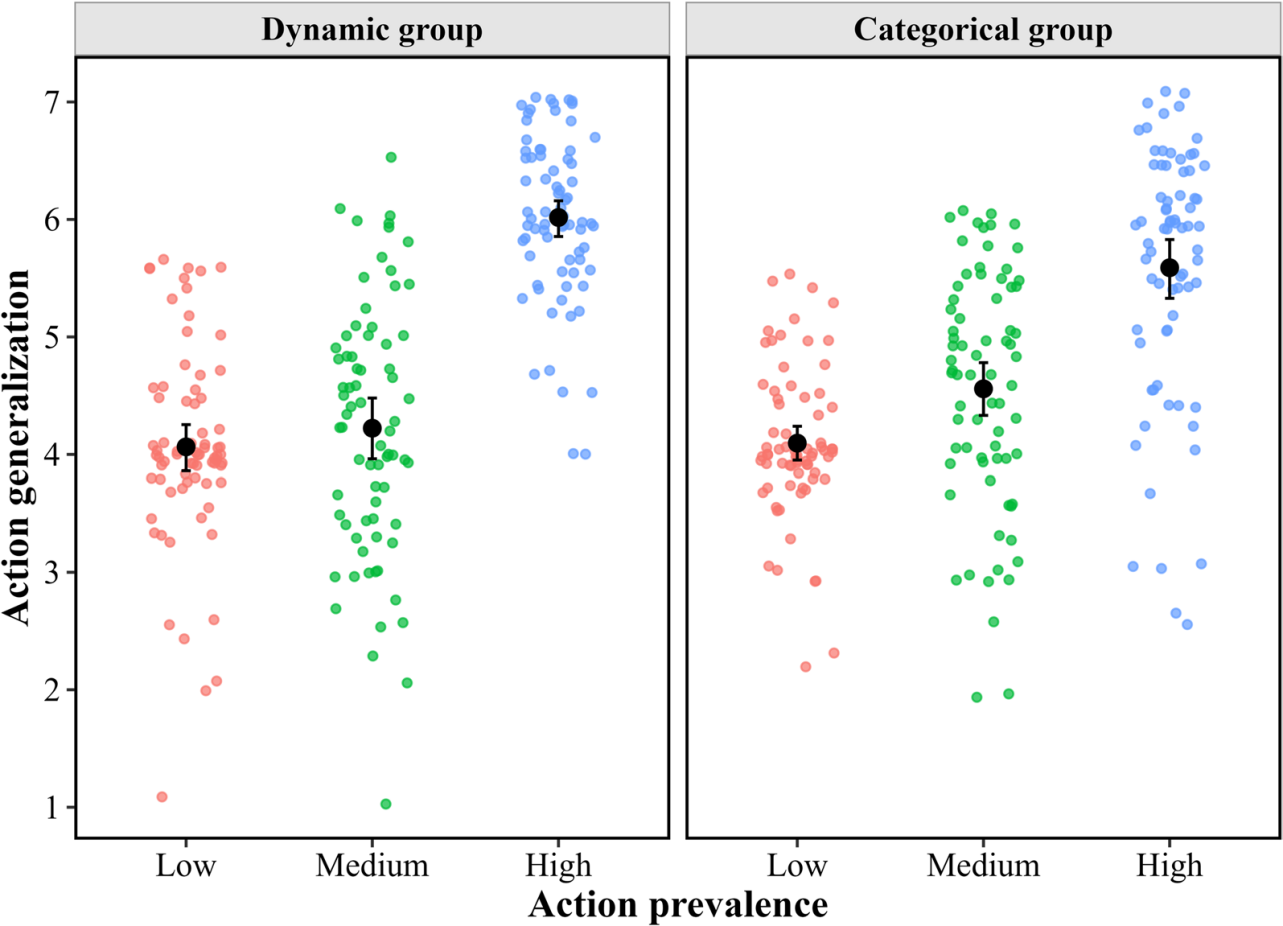
Action generalization as a function of action prevalence and group type

*Action Generalization* The mean action generalization index of each participant for each condition is shown in Fig. 3. Importantly, in the low prevalence condition, in which each action goal could be considered the generalized property, the rating value did not differ significantly from the mid-value (i.e., 4), confirming the baseline for both the dynamic (*t*(73*)* = 0.64, *p* = 0.525, Cohen’s *d* = 0.07, 95% CI of *d* = [− 0.15, 0.30]) and categorical (*t*(72*)* = 1.26, *p* = 0.213, Cohen’s *d* = 0.15, 95% CI of *d* = [− 0.08, 0.38]) conditions.

The 2 (group type as a between-subject factor: dynamic vs. categorical) × 3 (action prevalence as a within-subject factor: low vs. medium vs. high) analysis of variance (ANOVA) revealed that the main effect of group type was not significant (*F*(1, 145) = 0.04, *p* = 0.837, *η*_p_^2^ < 0.01); however, the main effect of action prevalence was significant (*F*(2, 290) = 170.43, *p* < 0.001, *η*_p_^2^ = 0.54). The post hoc comparisons indicated that the extent of action generalization in the high prevalence condition (*M* = 5.80, SE = 0.08) was greater than that in both the medium (*M* = 4.39, SE = 0.09; *t*(145*)* = 15.03, *p* < 0.001, Cohen’s *d* = 1.18, 95% CI of *d* = [0.99, 1.36]) and low (*M* = 4.08, SE = 0.06; *t*(145*)* = 16.69, *p* < 0.001, Cohen’s *d* = 1.43, 95% CI of *d* = [1.23, 1.64]) prevalence conditions. There was also a significant difference between the medium and low prevalence conditions (*t*(145*)* = 3.08, *p* = 0.008, Cohen’s *d* = 0.26, 95% CI of *d* = [0.06, 0.46]). The results indicate a graded increase in action generalization with the prevalence of the to-be-generalized action. This effect was modulated by the group type, as the interaction effect was significant (*F*(2, 290) = 7.46, *p* < 0.001, *η*_p_^2^ = 0.05).

The simple effects analysis showed that in the dynamic group condition, although the main effect of action prevalence was significant (*F*(2, 145) = 122.95, *p* < 0.001, *η*_p_^2^ = 0.63), the post hoc comparisons showed more action generalization in the high prevalence condition (*M* = 6.02, SE = 0.11) than in the medium (*M* = 4.22, SE = 0.12; *t*(145*)* = 13.55, *p* < 0.001, Cohen’s *d* = 1.49, 95% CI of *d* = [1.23, 1.76]) or low (*M* = 4.06, SE = 0.09; *t*(145*)* = 13.42, *p* < 0.001, Cohen’s *d* = 1.63, 95% CI of *d* = [1.33, 1.92]) prevalence conditions. However, there was no significant difference between the medium and low prevalence conditions (*t*(145*)* = 1.12, *p* = 0.799, Cohen’s *d* = 0.13, 95% CI of *d* = [− 0.16, 0.42]). The main effect of action prevalence was significant in the categorical group condition (*F*(2, 145) = 57.21, *p* < 0.001, *η*_p_^2^ = 0.44). However, the post hoc comparisons indicated a distinct pattern from that observed in the dynamic group condition. Specifically, the prevalent action in the high prevalence condition (*M* = 5.59, SE = 0.11) was more likely to be generalized to a new group member than in the medium (*M* = 4.56, SE = 0.12; *t*(145*)* = 7.73, *p* < 0.001, Cohen’s *d* = 0.86, 95% CI of *d* = [0.59, 1.13]) or low (*M* = 4.10, SE = 0.09; *t*(145*)* = 10.19, *p* < 0.001, Cohen’s *d* = 1.24, 95% CI of *d* = [0.95, 1.54]) prevalence condition, and the prevalent action in the medium prevalence condition was more likely to be generalized to a new group member than that in the low prevalence condition (*t*(145*)* = 3.23, *p* = 0.005, Cohen’s *d* = 0.39, 95% CI of *d* = [0.10, 0.67]).

### Experiment 2b

#### Methods

Since this experiment had a design that was similar to that of Experiment 2a but used different methods to manipulate shared-goal belief, we recruited a sample size that was similar to that of Experiment 2a. One hundred and fifty-one participants participated in the study and were paid approximately 5 RMB each for completing the investigation. Four participants were excluded due to incomplete investigations, and the final sample comprised 147 valid participants (57 men and 90 women, *M*_age_ = 19.80 years, SD = 1.78). The participants were randomly assigned to two groups, with 74 in the strong belief condition and 73 in the weak belief condition. All participants were of Han ethnicity, the majority ethnic group in China. A sensitivity power analysis indicated that this sample size provided 80% power to detect an effect with Cohen’s *d* ≥ 0.46 in different action generalization patterns between the two group types.

The procedure was almost the same as that of the dynamic group in Experiment 2a, except that before watching the videos, the participants were asked to read a text about how group members can behave (Henderson, 2009) to manipulate the strength of shared-goal belief. The text was presented above a screenshot from the beginning of the video that showed the two groups.

In the strong belief condition, the participants were told, “*In this picture above, the persons wearing the same color of vest are members of the same team. Humans, as social creatures, often interact with others to form groups or teams and frequently act together to achieve a collective goal. The group members usually act as a cohesive unit and engage in behaviors that help them move toward their shared goal. Although the group members behave in different ways, their actions are motivated by the same underlying intentions.*”

In the weak belief condition, the participants were told, “*In this picture above, the persons wearing the same color of vest are members of the same team. Although humans, as social creatures, often interact with others to form groups or teams, group members often pursue their own individual goals and may not necessarily act together to achieve a collective goal. The group members rarely act as a cohesive unit and engage in behaviors that help them move toward their own separate goals. The group members behave in different ways with different underlying intentions motivating their actions.*”

Hence, the design of this experiment was 2 (belief strength as a between-subject factor: strong vs. weak) × 3 (action prevalence as a within-subject factor: low vs. medium vs. high).

The participants were asked to report their ratings (*α* = 0.88) using the same scale as in the phase of measuring shared-goal belief for the dynamic group in Experiment 2a to ensure the validity of manipulating the strength of shared-goal belief. Then, the action generalization index was computed by averaging the mean ratings of both the food and drink scenes (*α* = 0.77), with items related to the likelihood of taking the minority’s food or drink being reverse-coded.

#### Results and discussion

*Validity of Shared-Goal Belief* An independent *t* test revealed that the belief that group members pursue a shared goal was greater for the strong belief condition (*M* = 5.21, SE = 0.10) than for the weak belief condition (*M* = 4.53, SE = 0.13; *t*(145*)* = 4.32, *p* < 0.001, Cohen’s *d* = 0.71, 95% CI of *d* = [0.37, 1.05]). These results suggest that the manipulation of shared-goal belief strength in this experiment is valid.

**Fig. 4:**
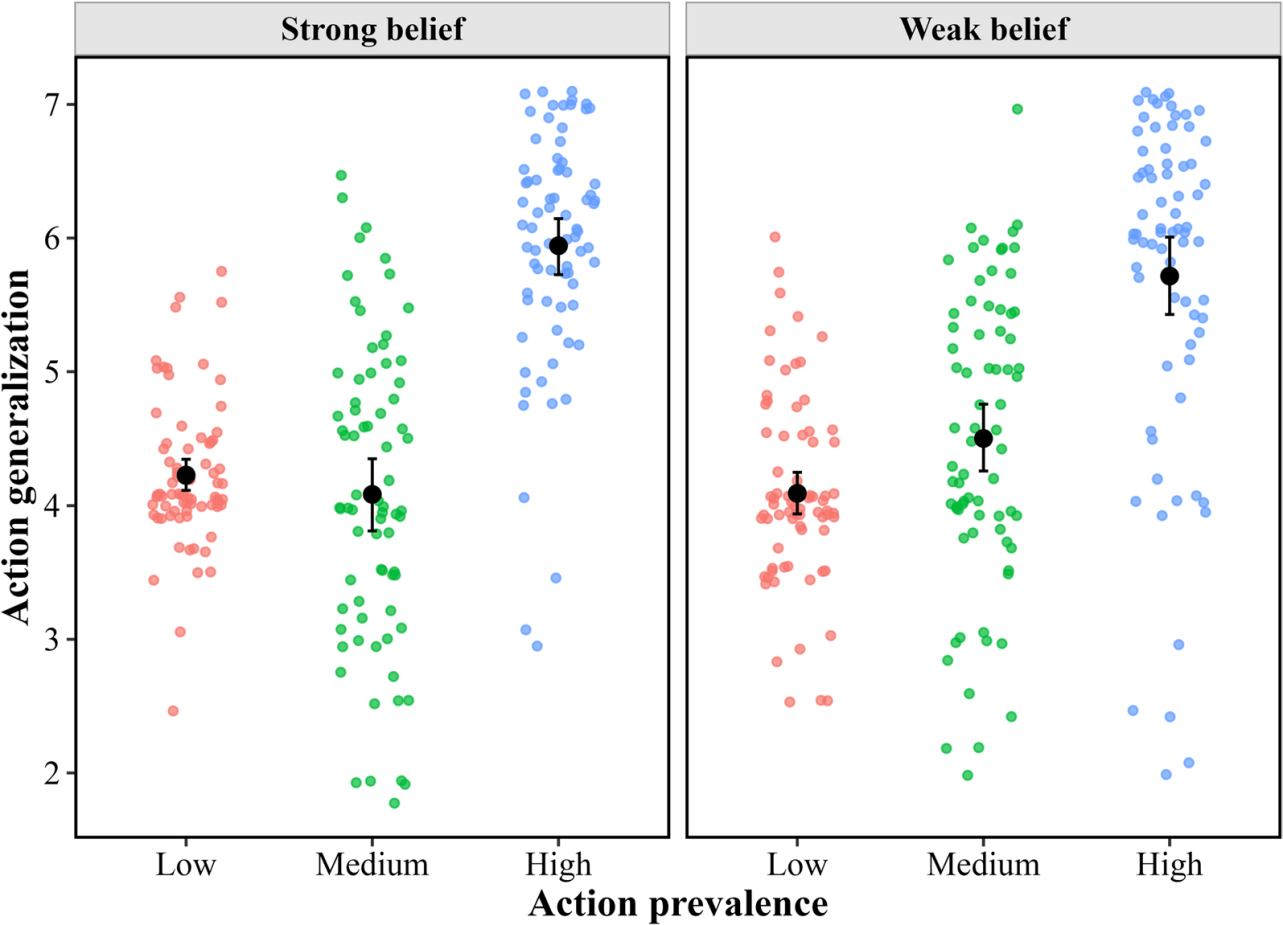
Action generalization as a function of action prevalence and belief strength

*Action Generalization* The mean action generalization index of each participant for each condition is shown in Fig. 4. In the low prevalence condition, where each goal could be treated as a generalized property, its rating value significantly differed from the mid-value (i.e., 4) in the strong belief (*t*(73*)* = 3.60, *p* < 0.001, Cohen’s *d* = 0.42, 95% CI of *d* = [0.18, 0.65]) but not in the weak belief (*t*(72*)* = 1.12, *p* = 0.268, Cohen’s *d* = 0.13, 95% CI of *d* = [− 0.10, 0.36]) conditions, partially confirming the baseline. This difference in the strong belief condition may be due to the shared-goal belief prompting the participants to infer a common group goal, leading them to perceive two distinct action-directed targets as highly likely. Despite this difference, the subsequent analysis still indicated a nongraded pattern in action generalization with increasing prevalence.

A 2 (belief strength as a between-subject factor: strong vs. weak) × 3 (action prevalence as a within-subject factor: low vs. medium vs. high) ANOVA revealed that the main effect of belief strength was not significant (*F*(1, 145) = 0.03, *p* = 0.856, *η*_p_^2^ < 0.01), but the main effect of action prevalence was significant (*F*(2, 290) = 147.62, *p* < 0.001, *η*_p_^2^ = 0.50). Post hoc comparisons suggested that the extent of action generalization in the high prevalence condition (*M* = 5.83, SE = 0.09) was larger than that in the medium (*M* = 4.29, SE = 0.09; *t*(145*)* = 13.59, *p* < 0.001, Cohen’s *d* = 1.18, 95% CI of *d* = [0.97, 1.39]) and low (*M* = 4.16, SE = 0.05; *t*(145*)* = 16.50, *p* < 0.001, Cohen’s *d* = 1.28, 95% CI of *d* = [1.09, 1.49]) prevalence conditions, and there was no significant difference between the medium and low prevalence conditions (*t*(145*)* = 1.21, *p* = 0.681, Cohen’s *d* = 0.10, 95% CI of *d* = [− 0.10, 0.31]). However, this effect was modulated by belief strength, as the interaction effect was significant (*F*(2, 290) = 5.16, *p* = 0.006, *η*_p_^2^ = 0.03).

The simple effects analysis indicated that in the strong belief condition, although the main effect of action prevalence was significant (*F*(2, 145) = 94.69, *p* < 0.001, *η*_p_^2^ = 0.57), the extent of action generalization in the high prevalence condition (*M* = 5.94, SE = 0.13) was greater than that in the medium (*M* = 4.08, SE = 0.13; *t*(145*)* = 11.66, *p* < 0.001, Cohen’s *d* = 1.43, 95% CI of *d* = [1.13, 1.72]) and low- (*M* = 4.23, SE = 0.07; *t*(145*)* = 12.03, *p* < 0.001, Cohen’s *d* = 1.32, 95% CI of *d* = [1.05, 1.58]) prevalence conditions in the post hoc comparisons, but there was no significant difference between the medium and low prevalence conditions (*t*(145*)* = 0.92, *p* = 1.000, Cohen’s *d* = 0.11, 95% CI of *d* = [− 0.18, 0.40]). In the weak belief condition, the main effect of action prevalence was significant (*F*(2, 145) = 66.81, *p* < 0.001, *η*_p_^2^ = 0.48), but the post hoc comparisons showed a different pattern from that of the strong belief condition. Specifically, the prevalent action in the high prevalence condition (*M* = 5.72, SE = 0.13) was more likely to be generalized to a new group member than that in the medium (*M* = 4.50, SE = 0.13; *t*(145*)* = 7.58, *p* < 0.001, Cohen’s *d* = 0.93, 95% CI of *d* = [0.63, 1.23]) or low (*M* = 4.09, SE = 0.07; *t*(145*)* = 11.30, *p* < 0.001, Cohen’s *d* = 1.24, 95% CI of *d* = [0.98, 1.51]) prevalence condition, and the prevalent action in the medium prevalence condition was more likely to be generalized to a new group member than that in the low prevalence condition (*t*(145*)* = 2.62, *p* = 0.029, Cohen’s *d* = 0.31, 95% CI of *d* = [0.02, 0.60]).

## General discussion

The current study investigated how the action prevalence among group members determines action generalization within the group, especially whether the belief that group members pursue a shared goal contributes to action generalization. The results showed that when a strong shared-goal belief was present and all group members exhibited the same goal-directed action, this action was more likely to be generalized to a new member. However, when group members pursued inconsistent goals, even as prevalence increased, action generalization did not increase, indicating a deviation from graded action generalization (i.e., nongraded action generalization). Moreover, Experiment 1 demonstrated that the more that the participants believed that group members pursued a shared goal, they were more likely to exhibit nongraded action generalization. In Experiment 2, experimental manipulation weakened the strength of the shared-goal belief, leading to a graded pattern of action generalization. Thus, consistent with our hypothesis, a shared-goal belief in social groups plays a crucial role in action generalization, at least for dynamic groups.

In this study, action prevalence was manipulated at three levels: low (50%), medium (75%), and high (100%), corresponding to the proportion of known members approaching the same external target. Importantly, in the low and medium prevalence conditions, the actions performed by the known group members were goal inconsistent, whereas in the high prevalence condition, the actions were goal consistent. These distinctions clarify that action generalization to a new group member occurs only when the actions of sampled members align with a consistent goal if the shared-goal belief is available. In such cases, the sampled evidence aligns with the shared-goal belief, which allows the to-be-generalized action to be identified as a group property (see Fig. 1). In contrast, when the shared-goal belief is absent, even when sampled members pursue inconsistent goals, increasing action prevalence from low to medium enhances the evidence for generalization, resulting in a graded relationship between the extent of action generalization and target action prevalence. Surely, expanding the group size and varying the range of action prevalence conditions could further strengthen the evidence for the role of the shared-goal belief in action generalization, especially if no differences in generalization extent are observed except when all members share the same goal. Nonetheless, the current findings highlight the critical influence of shared-goal belief in shaping action generalization.

Regarding the nongraded action generalization findings, one potential explanation is that participants might assign a superordinate or coordinative goal, with different individual-level goals, to anticipate a new group member’s possible action when the sampled group members exhibit different goal-directed actions. Consequently, both objects could be perceived as directed goals, resulting in a lack of action generalization for both low and medium action prevalence conditions. In the present paradigm, two items were set, and the participants were asked to rate the likelihood of behaving toward either of the two targets to avoid measuring only a specific type of individual goal-directed action and recognizing that the other action may also be generalized. These two items measured the likelihood of approaching each action goal (e.g., approaching an apple or a banana). In this case, if the participants expect that the group member will perform the prevalent action, theoretically, the higher rating for one item will mean a lower rating for the other item. Hence, the ratings for these two items should be negatively correlated. The negative correlation confirmed that participants were engaged during the task. Nevertheless, it is possible that the participants inferred a superordinate or coordinating goal encompassing different individual-level goals when group members pursued distinct actions. In this case, rather than the strong shared-goal belief weakening action generalization, the participants may have assumed a higher-order goal (e.g., collecting food) within the group. This argument would predict that the likelihood of collecting any target would not be correlational or be positively correlational, as either of the individual-level goals could be treated as achieving a superordinate goal. However, after all of the data showing the nongraded pattern were merged, a correlation between these two ratings was observed, with values of − 0.315 and − 0.668 in the low and medium prevalence conditions, respectively. The results suggest that as one specific goal became more expected, the other became less expected. Furthermore, the extent of generalization in the medium prevalence condition was restored when the shared-goal belief was weakened. However, the effect remained absent in the low prevalence condition (except in the strong belief condition of Experiment 2a, which may be attributed to the specific manipulation). This indicates that the participants treated the individual goals as distinct and did not assign a superordinate goal, supporting the interpretation of a specific goal-based generalization.

In addition, previous research has established that the belief that group members pursue a shared goal is closely linked to the perception of the group (Lickel et al., 2000) and significantly influences the expectation about the action of group members. Consistent with this finding, Experiment 2 revealed that although the mean extent of action generalization did not differ across group types or belief strength conditions, changes in the strength of shared-goal belief resulted in shifts in the pattern of action generalization. For example, when the shared-goal belief was weakened, the extent of action generalization gradually increased with the prevalence of actions and exhibited a graded pattern. These findings further highlight the pivotal role of shared-goal belief in shaping predictions about group actions.

Anticipating whether the unknown group member will perform a similar action to those observed emphasizes whether the target action is extended to a new instance, which falls under the domain of inductive generalization. The current findings align with the general domain of inductive generalization, demonstrating that knowledge of a property, such as how it is causally generated, impacts the extent of generalization (Rehder, 2009, 2017; Rehder & Hastie, 2001). Specifically, we found that people believe that members of dynamic groups pursue a shared goal and use this belief to guide their expectations about how group members should behave. Furthermore, our study challenges the notion of monotonic (graded) generalization, which suggests that positive observations increase the possibility of generalizing, whereas negative observations decrease it. We found that, consistent with previous research, special knowledge about observed properties can violate this pattern (Hayes & Heit, 2018; Vasilyeva & Lombrozo, 2020; Voorspoels et al., 2015). For instance, previous research has shown that nonmonotonic generalization can occur when people believe that observations are compiled by a helpful communicator (Voorspoels et al., 2015). Accordingly, our findings suggest that people do not simply treat social groups as boundaries for sampling actions but hold beliefs about how individuals belonging to the group perform actions. This belief primarily influences the extent of action generalization in the medium prevalence condition, where the accumulated evidence mostly conflicts with the available belief. This explains why no differences in the extent of action generalization were observed across group types or belief strength conditions in Experiment 2. However, in the low prevalence condition, the evidence does not ensure generalizability. Although we do not conclude that action generalization is always absent in the medium prevalence condition, we claim that its extent is lower than when shared-goal belief is absent or weakened. Overall, the results of this study indicate that action generalization is not solely determined by observed evidence; rather, it also depends on prior shared-goal belief.

Traditionally, stereotypes have been used to emphasize beliefs about specific social groups and have been suggested to be extended to unknown members, which often overlooks diversity and individuality (Chan et al., 2012; Delplanque et al., 2019; Greenwald & Banaji, 1995; Jussim et al., 2015; King et al., 2021). These beliefs share similarities with the current study, as both involve making probabilistic inferences about behavior in the absence of individual information. However, the emergence of stereotypes is influenced by various social processes, such as cultural transmission, media influence, and personal experiences (Chudnovskaya & Lipatova, 2018; Hutchison & Martin, 2015; Sczesny et al., 2019). Additionally, the prior belief that group members pursue a shared goal may be rooted in intuitive sociology, a natural and instinctive way of understanding group dynamics. Importantly, stereotypes are concrete beliefs about how group members behave, while the belief of group members pursuing a shared goal is a rule without specific knowledge about individual goals. From a hierarchical representation perspective, the shared-goal belief is located at a higher level than specific stereotypes representing knowledge about social groups. Within this framework, shared-goal belief constrains which attributes are generalized among group members (i.e., stereotypes). As a result, traits that support goal sharedness—such as competence in obtaining a specific apple—are more likely to be treated as stereotypical attributes. In turn, these attributes guide action generalization; for example, if competence in obtaining a specific apple is generalized to a new member, one may expect that this individual will also pursue this apple. Thus, the abstract belief in shared goals may activate stereotype-driven trait generalizations, which subsequently shape expectations about group members’ actions. Future research could explore the interaction between these two types of beliefs.

The findings from Experiment 2a support previous claims that people classify groups into different types and thereby hold different knowledge about these types (Lickel et al., 2006; Rutchick et al., 2008). Lickel and colleagues (2000; 2006) further classified dynamic and categorical groups and proposed that four basic group clusters should be identified: intimacy groups (e.g., families and friends), task groups (e.g., football teams and committees), social categories (e.g., Black people and women), and loose associations (e.g., people waiting for trains at stations) (Lickel et al., 2000, 2006). People use more complex, abstract, and enduring attributes to describe categorical or social-category groups, whereas they tend to use descriptions related to shared goals and motivations regarding dynamic or task groups. This regularity aligns with the fact that the dynamic group in Experiment 2a led to a nongraded increase in action generalization with action prevalence. Hence, the belief in the shared goals of group members may be more specific to dynamic or task groups. This account does not exclude the current conclusion that shared-goal belief contributes to action generalization; however, one should be cautious that the presence of this belief has a boundary. Furthermore, beyond differences in shared-goal belief, the inherent differences between dynamic and categorical groups—such as the tendency to infer trait-based similarity in categorical groups (Hamilton et al., 2011)—may have also contributed to the observed effects. These potential influences, including the cognitive representations activated by different group types (Rutchick et al., 2008), represent important avenues for future research aiming to disentangle the role of shared-goal belief from other group-related assumptions in guiding action generalization.

This study has several limitations. First, the participants were sampled from different universities and generally have a higher level of education; thus, it would be beneficial to replicate the results in a more diverse adult population to ensure generalizability. We anticipate that our results can be replicated in a diverse adult population, as a shared-goal belief is a common disposition ingrained in the social mind. Second, the small group size of the five members used in this study may have limited the cognitive processing of the groups. Considering that group size can impact group dynamics (Shen et al., 2014), it is important for future studies to explore the effects of larger group sizes. Third, although our results demonstrate that prior beliefs can qualitatively change the pattern of evidence-based generalization, the use of only three levels of action prevalence (low, medium, high) limits our ability to rigorously evaluate the precise functional form of the generalization curve—whether linear, curvilinear, or otherwise. Future studies should adopt finer-grained prevalence to characterize the precise functional form of generalization (e.g., linear, sigmoid) and validate the robustness of the observed effects. Fourth, the current study focused primarily on object-directed actions involving external goals (e.g., approaching a target). However, action goals can also be more abstract or internally driven—such as intending to help someone; these types of goals are typically referred to as intentions. Future research should investigate whether the conclusions of this study extend to more complex or intention-based actions.

Therefore, the belief that group members share goals plays a significant role in action generalization. Thus, social groups, particularly dynamic groups, not only define boundaries for selecting action evidence but also shape prior knowledge that supports action expectations. This phenomenon may originate from our intuitive understanding of social groups.

## Supplementary Information

Additional file 1.

## Data Availability

All data, analysis code, and research materials have been made publicly available via the Open Science Framework and can be accessed at https://osf.io/6ap74/?view_only=97741fd0566a41c49378f582e740abb7. None of the experiments were preregistered.

## Footnotes

1 The term “nongraded” is intended to describe findings that deviate from strict monotonicity, that is, cases where an increase in the prevalence of observed actions does not consistently lead to an increase in action generalization (i.e., where *x* > *y* does not always imply *f* (*x*) > *f* (*y*)).
