# Supplementary material for: Beyond evidence accumulation: shared-goal belief guides action generalization in social groups
Source: Cogn Res Princ Implic. 2025 Aug 26;10:56. doi: 10.1186/s41235-025-00666-x (PMC12380659; doi:10.1186/s41235-025-00666-x)
Supplement: Supplementary file 1 — Additional file 1. [file 41235_2025_666_MOESM1_ESM.docx]

**Supplementary Material**

**1. Ratings of participants’ expectations regarding the fifth person’ actions**

As two test items were used to measure action expectations—each corresponding to a distinct action directed toward a different target—participants’ ratings of which action the fifth person was expected to perform in the low prevalence condition were compared across various experiments and scenarios. Non-significant differences between the ratings of the two items support the rationale for randomly determining the prevalent action in the low-prevalence condition.

In Experiment 1, there was no difference between ratings for the two items in Scenario 1 involving taking food (*t*(102) = -1.97, *p* = 0.051, Cohen’s *d* = -0.19, 95% CI of *d* = [-0.01, 0.01]); similarly, no difference was found in Scenario 2 involving taking a drink (*t*(102) = 0.19, *p* = 0.849, Cohen’s *d* = 0.02, 95% CI of *d* = [-0.18, 0.21]).

In Experiment 2a, for the dynamic group condition, there was no difference between ratings for two items (Scenario 1: *t*(73) = 0.26, *p* = 0.794, Cohen’s *d* = 0.03, 95% CI of *d* = [-0.20, 0.26]; Scenario 2: *t*(73) = -1.28, *p* = 0.203, Cohen’s *d* = -0.15, 95% CI of *d* = [-0.38, 0.08]). The same was found for the category group condition (Scenario 1: *t*(72) = -1.05, *p* = 0.296, Cohen’s *d* = -0.12, 95% CI of *d* = [-0.36, 0.11]; Scenario 2: *t*(72) = -1.02, *p* = 0.309, Cohen’s *d* = -0.12, 95% CI of d = [-0.35, 0.11]).

In Experiment 2b, for the strong belief condition, there was no difference between ratings for two items in Scenario 1 (*t*(73) = -0.29, *p* = 0.772, Cohen’s *d* = -0.03, 95% CI of *d* = [-0.27, 0.20]), while a significant difference was observed in Scenario 2 (*t*(73) = -4.08, *p* < 0.001, Cohen’s *d* = -0.47, 95% CI of *d* = [-0.71, -0.24]). There was no difference for the weak belief condition (Scenario 1: *t*(72) = -1.56, *p* = 0.123, Cohen’s *d* = -0.18, 95% CI of *d* = [-0.42, 0.05]; Scenario 2: *t*(72) = -0.10, *p* = 0.921, Cohen’s *d* = -0.01, 95% CI of d = [-0.24, 0.22]).
